# Sports injuries profile of a first division Brazilian soccer team: a descriptive cohort study

**DOI:** 10.1590/bjpt-rbf.2014.0120

**Published:** 2015-10-06

**Authors:** Guilherme F. Reis, Thiago R. T. Santos, Rodrigo C. P. Lasmar, Otaviano Oliveira, Rômulo F. F. Lopes, Sérgio T. Fonseca

**Affiliations:** 1 Departamento Médico, Clube Atlético Mineiro, Belo Horizonte, MG, Brazil; 2 Programa de Pós-Graduação em Ciências da Reabilitação, Universidade Federal de Minas Gerais (UFMG), Belo Horizonte, MG, Brazil

**Keywords:** epidemiology, sport, incidence, soccer injuries, physical therapy

## Abstract

**Objective:**

To establish the injury profile of soccer players from a first division Brazilian soccer team. In addition, we investigated the association between the characteristics of the injuries and the player's age and position.

**Method:**

Forty-eight players from a Brazilian first division soccer team were followed during one season. Descriptive statistics were used to characterize the injury profile. Spearman's tests were used to verify the association between the number and severity of injuries and the player's age. Chi-square test was used to verify the association between type of injury and player's position. Fisher's exact test was used to verify the association between the severity of injuries and player's position.

**Results:**

The incidence of injuries was 42.84/1000 hours in matches and 2.40/1000 hours in training. The injury severity was 19.5±34.4 days off competition or training. Lower limb was the most common location of injury and most injuries were muscular/tendinous, overuse, non-recurrent, and non-contact injuries. Player's age correlated with the amount and severity of muscle and tendon injuries. Defenders had more minimal injuries (1-3 days lost), while forwards had more moderate (8-28 days lost) and severe injuries (>28 days lost). Furthermore, wingbacks had more muscle and tendon injuries, while midfielders had more joint and ligament injuries.

**Conclusion:**

The injury profile of the Brazilian players investigated in this study reflected regional differences in soccer practices. Results confirm the influence of the player's age and position on the soccer injuries profile.

## Introduction

Professional soccer requires a high level of financial investment in structure and maintenance^1^^,^ 2. Several studies have identified financial losses associated with high number of injuries in soccer due to the withdrawal of players from matches^1^^-^ 4. The implementation of preventive measures to reduce soccer injuries has received attention from sports physical therapists^5^. Documentation of the incidence, severity, and nature of injuries in high-level professional sports is the first step in developing effective preventive strategies^6^. The occurrence of soccer injuries may be affected by sport-specific and context-specific factors. Therefore, in order to understand the process of soccer injury, we must take into consideration not only the characteristics of the players and their roles, but also the characteristics of the place where the sport is played.

Context-specific factors such as number of matches during a season, weather and, style of play may distinctly affect the nature and incidence of soccer injuries in different countries^2^^,^ 7. For example, Waldén et al.^2^ observed that the risk of injuries in matches was significantly greater in English and Dutch teams than in teams from France, Italy, and Spain. Despite the absence of comparative studies, Brazilian soccer players are known to have a different style of playing compared to European players. For example, Brazilian players have a more free-flowing offensive style with more individual plays compared to European players. Unfortunately, few studies have investigated injury profile in Brazilian professional soccer^8^^,^ 9. The most recent study was conducted with a second division Brazilian soccer team^9^. However, the profile of injury found by this study may be not applicable to first division teams given that Emery et al.^10^observed a higher incidence of injuries in first division teams compared to other ones. Thus, the establishment of the injury profile of a Brazilian first division soccer team can contribute to the understanding of the factors that have to be considered during the implementation of preventive strategies.

Sport-specific factors, such as the player's age and position on the soccer field, may also affect the injury profile. Coelho et al.^11^ observed that the efforts during matches varied according to the field position. Specifically, wingbacks exerted a higher amount of maximum efforts than other players^11^. These authors also observed that midfielders did not participate in as many maximum efforts as the other players^11^. The different amount of efforts performed by players according to the field position may also influence the soccer injury profile. Another factor that may influence the soccer injury profile is the athlete's age. Arnason et al.^12^investigated athletes between 16 and 38 years of age and found that the older the player is, the greater the chance of injury. The age factor has been a new focus in soccer studies given the observed increase in soccer players over 30 in professional teams^13^.

Sport- and context-specific factors should be considered in studies that aim to investigate soccer injury profiles thoroughly. Accordingly, the aim of this study was to establish the injury profile of soccer players from a first division Brazilian team. In addition, a secondary aim was to investigate the characteristics of injuries according to age and field position. This study can enhance the understanding of injuries in soccer players and provide knowledge that can help sports physical therapists to design programs focusing on soccer injury prevention.

## Method

### Subjects and Experimental Design

This descriptive cohort study was conducted with players from a Brazilian first division soccer team who were followed during one season. The players were considered eligible for this study if they did not Table 1 **.** Characteristics of the athletes according to field position. have any musculoskeletal injury before the beginning of the study. The medical staff was responsible for screening out players with musculoskeletal injuries. This study was an open cohort, thus if a player joined or left the team during the season, he was not excluded from the investigation, but the number of days that he was followed were considered in the descriptive and inferential analysis. Thirty-eight male athletes were evaluated, and one athlete was excluded due to injury. Thus, 37 athletes were initially followed. During the season, 12 new athletes joined the team, and none had any musculoskeletal injury. In addition, 13 athletes left the team during the season and consequently were not followed for the entire season. At the end of the season, this study investigated 48 male athletes for 238.3±103.2 days, including 4 goalkeepers, 6 defenders, 7 wingbacks, 20 midfielders, and 11 forwards. The characteristics of this sample are shown in Table 1. All athletes signed an informed consent form. This study was approved by the Research Ethics Committee of Universidade Federal de Minas Gerais (UFMG), Belo Horizonte, MG, Brazil (ETIC 0493.0.203.000-09). 

**Table 1.** Characteristics of the athletes according to field position.

**Field Position**	**Age (** ***years*** **)**	**Body Mass (** ***kg*** **)**	**Height (** ***cm*** **)**	**BMI (** ***kg/m*** **^2^** **)**
Goalkeeper (*n*=4)	21.8 (2.2)	89.97 (2.12)	188.50 (3.19)	25.33 (0.85)
Defender (*n*=6)	24.8 (4.6)	85.52 (4.39)	187.50 (5.33)	24.33 (1.07)
Wingback (*n*=7)	25.1 (5.1)	73.03 (1.72)	175.50 (2.12)	23.72 (0.85)
Midfielder (*n*=20)	25.4 (4.5)	74.83 (8.51)	176.82 (6.06)	23.79 (1.76)
Forward (*n*=11)	26.4 (4.8)	78.00 (8.08)	176.44 (4.40)	25.01 (8.08)
Total (*n*=48)	25.2 (4.5)	78.73 (8.51)	179.69 (7.01)	24.35 (1.58)

Age, body mass, height and body mass index (BMI) are shown as mean (standard deviation).

### Procedures

Injury recording followed the guidelines for injury definitions and data collection procedures in studies on soccer injuries of *Fédération Internationale de Football Association* (FIFA) *Medical Assessment and Research Centre* (F-MARC)^14^. Injury was defined as any physical complaint that results in a player being unable to take part in at least one subsequent soccer training session or match^14^. Injuries were recorded by the physical therapy team of the club, who was trained to use the F-MARC form at the beginning of the season. The injury event was recorded immediately after it occurred, and the match and training hours were recorded by the team's physiologist.

Injury recording considered the moment at which the injury occurred (match or training), as well as severity, location, type, mechanism and recurrence. Injury severity was defined according to the number of days lost by the player between the day of the injury and the return to full participation in team training, and the availability to be selected to play^14^. Injury severity was also classified according to the number of days lost: minimal (1-3 days), mild (4-7 days), moderate (8-28 days), and severe (>28 days)^14^. Location of injury was defined according to the following categories: head/neck, upper limbs, trunk, and lower limbs^14^. The type of injury was classified as fracture/bone stress, joint (non-bone)/ligament, muscle/tendon, contusions, laceration/skin injury, central/peripheral nervous system, and others^14^. All muscle strains were confirmed by diagnostic imaging. The mechanism of injury was classified as traumatic, i.e. resulting from a specific and identifiable event, or as overuse, i.e. caused by repeated micro-traumas, even without a simple and identifiable event^14^. Recurrence was defined as the same type and site of injury recorded in the same season, and that occurred after the player returned to full participation in soccer^14^. Recurrence was classified as early, when the injury occurred at an interval less than two months; or as late, when it occurred between 2 and 12 months, following return to full participation in soccer^14^.

### Data preparation

Injuries were organized in number and percentage according to location, type, mechanism, recurrence, and whether they occurred with or without contact. The incidence of injury during matches and training was reported as the number of injuries per 1000 hours played. Injury severity was shown both in relation to the mean and standard deviation of lost days, as well as quantity and percentage of injuries according to the severity classification. The quantity and severity (lost days) of injuries were divided by the number of days that each player was monitored. Since a player could enter or leave the team during the study, this normalization procedure was chosen in order to consider differences of monitoring-time of the players in the quantity and severity (lost days) of injuries.

### Statistical analyses

Descriptive statistics were done in order to characterize the injury profile. Inferential statistics were performed to investigate the characteristics of injuries according to age and field position. Tests of association were chosen to investigate the association between the player's age and the characteristics of injury. Initially, the assumption of normality was verified using the Shapiro-Wilk test, which revealed that these variables were non-normally distributed. Considering this, Spearman's test was used to verify the association between the player's age and a) normalized total number of injuries, b) normalized number of muscle/tendon injuries, and c) normalized number of joint/ligament injuries. Spearman's test was also used to verify the association between the player's age and a) normalized severity of the total group of injuries, b) normalized severity of muscle/tendon injuries, and c) normalized severity of joint/ligament injuries.

Since the player's field position is a categorical variable, chi-square and Fisher's exact test were chosen to investigate the relationship between the player's position and the characteristics of injury. The chi-square test was chosen to verify the association between player's position and type of injury since the expected frequency in each cell of the contingency table was greater than 5. Fisher's exact test was chosen to verify the association between the player's position and the classification of severity of injuries since the expected frequency in some cells of the contingency table was lower than 5. Furthermore, Cramer's V test was used to calculate the effect size for both the chi-square and Fisher's exact test. The interpretation of Cramer's V test output considered that the closer the value is to 1, the greater the effect size. In addition, when a significant association was found, analysis of adjusted residuals was used to verify which subgroups contributed most to the result. A significant adjusted residual indicates that the cell of the contingency table made a significant contribution to the main statistic. Since the adjusted residual is a z score, if the value lies outside of ±1.96, it indicates that the number of cases in that cell of the contingency table is different than expected, considering *p*<0.05. Positive adjusted residuals indicate that the cell is over-represented in the sample compared to the expected frequency and negative residuals indicate that the cell is under-represented in the sample compared to the expected frequency. The level of significance (α) was set at 0.05 for all inferential analyses.

## Results

The season consisted of 334 days, during which 58 matches were played. The number of matches per athlete was 24±15.3, and the training time per athlete was 315±54.8 hours. Seventeen players (3 goalkeepers, 2 defenders, 9 midfielders, and 3 forwards) did not sustain any injuries, and 31 players (1 goalkeeper, 4 defenders, 7 wingbacks, 11 midfielders, and 8 forwards) sustained some type of injury. Forty-one injuries (0.71 injuries per match) occurred during matches (58.6%), and 29 injuries occurred during training (41.4%). The incidence of injury was 42.84/1000 hours of matches and 2.40/1000 hours of training. The mean of severity of injury was 19.5±34.4 lost days. All injuries occurred in the lower limbs and their characteristics are presented in Table 2. In addition, the distribution of type of injury according to the classification of severity is presented in Table 3. The anatomic location of the strains and sprains is presented in Table 4. 

**Table 2.** Characteristics of injuries.

	**Number**	**%**
Mechanism Traumatic	15	21.4
Overuse	55	78.6
Recurrence Non-recurrent	65	92.9
Early	3	4.3
Late	2	2.9
Contact	14	20.0
Non-contact	56	80.0

The percentages in each characteristic are related to the total number of injuries (70).

**Table 3.** Distribution of injury types according to classification of severity.

**Injury type**	**Classification of Severity**			
	**Minimal** **(1-3 days)**	**Mild** **(4-7 days)**	**Moderate** **(8-28 days)**	**Severe** **(>28 days)**	**Total**
Muscle and tendon	6 (14.6%)	9 (22.0%)	20 (48.8%)	6 (14.6%)	41 (58.6%)
Joint and ligament	9 (36.0%)	4 (16.0%)	7 (28.0%)	5 (20.0%)	25 (35.7%)
Fracture and bone stress	1 (50.0%)	0 (0%)	1 (50.0%)	0 (0%)	2 (2.9%)
Central/peripheral nervous system	0 (0%)	1 (100%)	0 (0%)	0 (0%)	1 (1.4%)
Others	0 (0%)	1 (100%)	0 (0%)	0 (0%)	1 (1.4%)
Total	16 (22.9%)	15 (21.4%)	28 (40.0%)	11 (15.7%)	70

Percentages are related to the total cases of each row, except the row totals and column totals, which percentages are related to the total amount of injuries.

**Table 4.** Anatomic location of strains and sprains.

	**Number**	**%**
Muscles strained	19	100
Rectus femoris	9	46.37
Hip adductors	5	26.32
Hamstring	3	15.79
Iliopsoas	2	10.53
Joints sprained	24	100
Ankle	12	50.0
Knee	12	50.0

**Figure 1.**
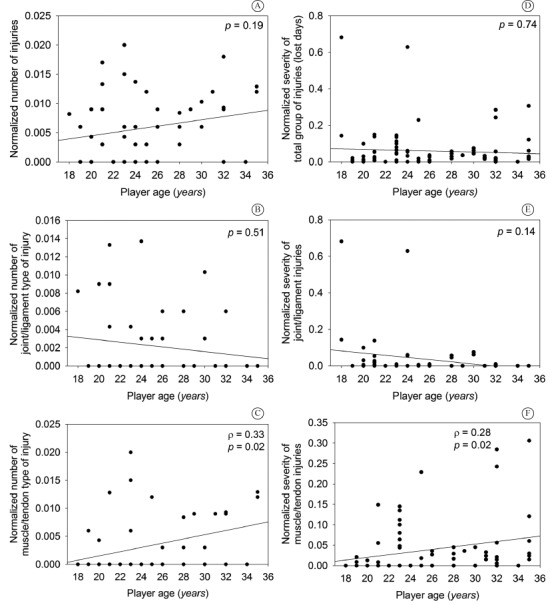
Scatter plot of each correlation investigated.

The investigated associations are shown in Figure 1. Spearman's test showed no evidence of association between player age and total number of injuries (*p*=0.19) or between age and number of joint/ligament injuries (*p*=0.51). However, a positive association was observed between age and number of muscle/tendon injuries (ρ=0.33, *p*=0.02). In relation to severity, no association was observed between player age and severity of total injuries (*p*=0.74) or between age and severity of joint/ligament injuries (*p*=0.14). However, an association was observed between age and severity of muscle/tendon injuries (ρ=0.28, *p*=0.02). 

The goalkeepers were not considered in the investigation of the association between field position and type of injury, because they experienced no muscle/tendon or joint/ligament type of injuries. The chi-square test showed an association between field position and type of injury (χ^2^ (3) = 10.45, *p*=0.02). Cramer's V test showed that this association had a magnitude of 0.40 (*p*=0.02). The analysis of adjusted residuals showed that the midfielders had the greatest association between field position and joint/ligament type of injury (Z=2.1), while wingbacks had the lowest association (Z=-2.1). Furthermore, the wingbacks showed the greatest association between field position and muscle/tendon type of injury (Z=2.1), while midfielders had the lowest association (Z=-2.1).

Fisher's exact test showed an association between field position and classification of severity (*p*<0.01). Cramer's V test showed a correlation with magnitude of 0.36 (*p*<0.01). The analysis of adjusted residuals showed that defenders contributed more to the association between field position and minimum injury severity (Z=2.2), while the forwards contributed less to this association (Z=-2.5). Furthermore, the forwards contributed more to the association between field position and moderate injury severity (Z=4.3), while wingbacks (Z=-2.2) and defenders (Z=-2.0) contributed less to this association.

## Discussion

This study characterized the injury profile of a first division Brazilian Championship team during one season. The characteristics of this injury profile may be explained by sport- and context-specific factors. The injury incidence observed during matches (42.84/1000 hours) was greater than the injury incidence reported by the Union of European Football Associations (UEFA) teams (mean of 27.5/1000 hours)15 and by the Japanese first division teams (mean of 21.8/1000 hours)16. A comparison with other Brazilian studies was not possible due to methodological differences, such as the definition of sports injury and incidence rate8,9. The incidence of injury (per 1000 h) during matches found in this study was nearly 18 times greater than that observed during training. The greater incidence of injury during matches has already been shown in other studies^2^^,^ 17. This may be related to the greater competitive pressures on the players during games as opposed to during training, and thus, this can be considered a sport-specific factor^18^. It is noteworthy that the eighteen-fold relationship found in this study is four to six times greater than the mean relationship reported in other studies^19^. This difference may be related to the number of matches played during a season^2^. The mean matches per season for UEFA teams is 32^15^; and ranges from 30-44 for Japanese league teams^16^. This differs greatly from the 58 matches played by the team investigated in this study. This suggests that context-specific factors, such as the number of matches per season, can explain differences in the profile of soccer injuries observed in different countries. It is important to highlight that the design of this study does not allow the definition of the causes of the observed injuries.

The majority of injuries were classified as of moderate severity. The severity found (19.5±34.4 lost days) in this study is almost the same as reported in other studies^4^^,^ 17. In addition, all injuries occurred in the lower limbs. Muscle/tendon injuries, followed by joint/ligament, were the most common types of injury. Strain injuries predominated among the muscle/tendon injuries, whereas sprain injuries predominated among joint/ligament injuries. These characteristics are in agreement with other studies^15^^-^ 17. Furthermore, most injuries occurred due to overuse (78.6%). This mechanism of injury is possibly related to the type of demand placed on the musculoskeletal system by soccer practice, and thus a sport-specific factor^20^. For example, during kicking or cutting maneuvers, the musculoskeletal system has to store part of the elastic energy for subsequent reuse^20^. This mechanism allows less muscle overload during matches and training. The presence of some biomechanical factor related to the soccer player that interferes with the elastic return capability of the musculoskeletal system (i.e. muscle weakness) may result in greater stress on the tissues, which increases the potential for injury. Therefore, the characteristics of severity, type, and mechanism of injury found in the investigated team may be considered as sport-specific factors.

Most of the injuries found in this study were non-recurrent and non-contact. The recurrence rate (7.1%) was lower than what is reported in the literature (20-25%)^19^. The predominance of non-contact injuries is reported in other studies and can be related to the higher rate of overuse injuries compared to traumatic injuries^17^^,^ 18. However, the non-contact injury rate found in this study (80%) is higher than the rates reported in the literature^17^^,^ 18. This high rate suggests the existence of a high demand placed on the musculoskeletal system during soccer actions^18^^,^ 20. Activities such as running and cutting are not only related to the mechanism and type of injury (overuse and muscle/tendon types), but may also help explain the large number of non-contact injuries^18^^,^ 20. Thus, the predominance of non-contact injuries reinforces the importance of implementing preventive programs for soccer players.

The age of the player was positively associated with the quantity and severity of muscle/tendon type of injury. Age has already been previously identified as a risk factor for the development of injuries^12^. The association found in the present study may be related to physiological factors^21^^,^ 22 that reduce the capability of the musculoskeletal system to deal with stress. Muscle mass in adulthood decreases progressively with age, due to the reduction in the amount and cross-sectional area of muscle fibers^21^. In addition, tendons lose their capacity to store, return, and transmit energy throughout life^22^. This phenomenon is more intense in older individuals^21^, which may have contributed to the observed association of the player's age to the quantity and severity of injuries. Another factor that could explain this association is the occurrence of prior injuries^23^. Considering that the muscle/tendon injuries are frequent, older players may have suffered this type of injury during other seasons, which could affect the tissue structure and predispose them to injury recurrence. Future cohort studies should be developed to investigate tissue changes in professional soccer players, in order to identify the role of these factors for injury development. Independent of causal factors, preventive programs and recovery programs after matches should consider different activities according to the player's age.

The player's field position influenced the type and the severity of injury. Wingbacks had more muscle/tendon injuries, while midfielders had more joint/ligament injuries. Wingbacks have been reported to perform maximal efforts during matches by means of higher number of sprints^11^^,^ 24. This may be related to tactical patterns of modern soccer, characterized by wingbacks performing defensive and attacking roles in short periods of time^24^. This may overload muscle and tendon tissues, which could predispose the wingbacks to injuries. The greater number of joint/ligament-type injuries associated with midfielders could be related to their role of linking defense and attack actions. 

This role requires frequent turning and changing of direction, which increases the demands on the joints and ligaments^20^. Furthermore, this study observed that defenders had more minimal severity injuries, while forwards had more moderate and severe injuries. Di Salvo et al.^24^ reported that defenders perform fewer sprints during matches and, thus, face fewer physical demands compared to other field positions. This may be related to the lesser severity of the injuries observed in defenders. Moreover, the more moderate injuries observed in forwards could be related to the higher demand for sprints performed by this field position, together with the wingbacks. Another factor that could have contributed to this association was the fact that the investigated forwards tended to be slightly older than the players of other positions. Despite this, the injury profile may be more strongly related to different demands of motions required by the athlete's position on the field.

The turnover of athletes during the season could be considered a limitation of this study. However, due to the difficulty of following an athlete after leaving, this study controlled for this limitation by weighting the analyses according to the days played by each athlete. The investigation of just one season could also be considered a limitation, nevertheless other studies reported no differences in the injury profiles in different seasons^23^^,^ 25. Furthermore, this study investigated athletes from one team and, thus, features of this team may affect the results. It is noteworthy that the investigated team plays in the first division Brazilian Championship and offers an infrastructure similar to other teams of this division. In addition, other factors not considered by this study may also influence the injury profile, such as training characteristics and previous injuries. Finally, the results of this descriptive cohort can help in the design of future epidemiological studies, such as analytical studies with multiple soccer teams, that could consider the factors investigated in the present study.

## Conclusion

This study described the injury profile of soccer players from a first division Brazilian team. This profile had similarities and differences with other reported profiles of teams from other countries, which may reflect the influence of both sport- and context-specific factors for the development of soccer injury. The quantity and severity of injuries were associated with the player's age and field position. The results of this study can enhance the knowledge of injuries in Brazilian soccer and help sports physical therapists to plan preventive programs.
